# Strain Field Around Individual Dislocations Controls Failure

**DOI:** 10.1002/smtd.202400654

**Published:** 2024-09-06

**Authors:** Christoph Gammer, Inas Issa, Andrew M. Minor, Robert O. Ritchie, Daniel Kiener

**Affiliations:** ^1^ Erich Schmid Institute of Materials Science Austrian Academy of Sciences Jahnstrasse 12 Leoben A‐8700 Austria; ^2^ Department Materials Science Chair of Materials Physics Montanuniversität Leoben Jahnstrasse 12 Leoben A‐8700 Austria; ^3^ National Center for Electron Microscopy Molecular Foundry Lawrence Berkeley National Laboratory Berkeley CA 94720 USA; ^4^ Department of Materials Science & Engineering University of California Berkeley CA 94720 USA; ^5^ Materials Sciences Division Lawrence Berkeley National Laboratory Berkeley CA 94720 USA

**Keywords:** 4D STEM strain mapping, fracture experiments, in situ TEM, nanomechanical testing

## Abstract

Understanding material failure on a fundamental level is a key aspect in the design of robust structural materials, especially for metals and alloys capable to undergo plastic deformation. In the last decade, significant progress is made in quantifying the stresses associated with failure in both experiments and simulations. Nonetheless, the processes occurring on the most essential level of individual dislocations that govern semi‐brittle and ductile fracture are still experimentally not accessible, limiting the failure prediction capabilities. Therefore, in the present work, a one‐of‐a‐kind nanoscale fracture experiment is conducted on a single crystalline Cr bending beam in situ in the transmission electron microscope and for the first time quantify the transient strains around individual dislocations, as well as of the whole dislocation network during crack opening. The results reveal the importance of both pre‐existing and newly emitted dislocations for crack‐tip shielding via their intrinsic strain field and provide guidelines to design more damage tolerant materials.

## Graphical abstract

By conducting in situ transmission electron microscopy strain mapping during a nanoscale fracture experiment, the dislocation processes taking place at the crack tip in the rather brittle metal chromium is detailed. Moreover, for the first time, the local stress fields of the dislocations shielding the crack is directly measured.

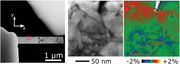

## 1 Introduction

Understanding failure of materials is of central importance for their potential application. Therefore, there has been significant work focused on predicting fracture,^[^ ^1^, ^2^ ^]^ that is typically divided into brittle, semi‐brittle and ductile fracture.^[^ ^3^, ^4^ ^]^ Purely brittle fracture is associated with breaking of bonds at the crack‐tip to create a new fracture surface, evidenced from a linear‐elastic stress–strain curve, as originally described by the well‐known Griffith model.^[^ ^5^ ^]^ Here the fracture toughness, *K_c_* _≈_ (γ *E*)^½^, where *E* is Young's modulus and γ the fracture energy, which can be taken as the surface energy for ideally elastic fracture.^[^ ^6^, ^7^ ^]^ In contrast, the fundamental notion of ductile fracture starts with dislocation emission from a crack tip, which serves to induce crack‐tip blunting,^[^ ^6^ ^]^ followed by more extensive plasticity to enable fracture mechanisms such as microvoid coalescence. The fracture toughness can be considered in terms of the stress intensity for crack‐tip dislocation emission, *K_e_*_≈_ (γ_usf_ *E*)^½^, where γ_usf_ is the unstable stacking‐fault energy for shear.^[^ ^7^ ^]^ Accordingly, predicting ductile fracture is more of a challenge.^[^ ^8^, ^9^ ^]^ The reason is that materials prone to semi‐brittle and ductile failure show an increasingly more complex nonlinear stress–strain curve stemming from different energy dissipating plastic deformation mechanisms, naturally in metals primarily associated with dislocations. It is well known that dislocation plasticity leads to crack‐tip shielding, and hence enhances fracture toughness. What is currently lacking, however, is knowledge to the extent that shielding is a result of the dislocation plasticity blunting the crack, or the local strain fields from the emitted dislocations counteracting the applied stress. Notably, this capability to identify and quantify the fundamental toughening mechanisms is crucial for more focused material development, or to better understand and harness recently reported fatigue crack healing in nanostructured metals.^[^ ^10^ ^]^

To date, atomistic simulations have been the main methodology used to link crack‐tip plasticity to dislocation generation and crack‐tip shielding;^[^ ^11^ ^]^ as such, it has been suggested that pre‐existing dislocations play an important role.^[^ ^12^ ^]^ Still, when using atomistic simulations alone, we face limitations such as the requirement of accurate interatomic potentials for complex alloys, as well as limitations in model size and strain rate, respectively. Therefore, there is demand for direct quantitative experimental measurements on the nanoscale. Specifically, the interplay between the complex stress field of dislocations with the highly inhomogeneous stress state at a crack‐tip is known to play an important role, but at the same time poses an immense experimental challenge. Even though progress has been made to map stress‐fields at more local scales^[^ ^13^ ^]^ and to measure the mechanical response of the confined volumes surrounding the crack via small‐scale deformation specimens more accurately,^[^ ^14^ ^]^ the quantitative measurement of the nanoscale dynamic stress‐field at the crack‐tip is still lacking. Fundamentally, knowledge of the strain field around defects during operation and failure is not only of importance for understanding the fracture of nanoscale components, but also for understanding the influence of atomistic structural imperfections on the fracture properties of advanced structural materials,^[^ ^15^ ^]^ as well as the impact of structural defects on functional properties,^[^ ^16^ ^]^ such as irradiation degradation^[^ ^17^ ^]^ or hydrogen storage/embrittlement.^[^ ^18^ ^]^

Experimentally mapping the interaction of dislocations with any stress field has been a challenge, as it simultaneously requires nanoscale resolution for a large field of view, a sufficiently thick specimen and dynamic in situ deformation capabilities. While it has been demonstrated that postmortem transmission electron microscopy (TEM) investigations can be carried out after fracture,^[^ ^19^, ^20^ ^]^ they are not sufficient to describe the dynamic evolution of dislocation configurations at the nanoscale, as they represent a transient state. In situ synchrotron measurements could sample large volumes, but lack the required resolution.^[^ ^21^ ^]^ Therefore, in the present manuscript we use four‐dimensional scanning TEM (4D‐STEM), enabling us to directly measure local transient strain‐fields by recording a map of nanobeam electron diffraction (NBD) patterns.^[^ ^22^ ^]^ Notably, we make use of a unique setup combining a direct electron detector operating in electron counting mode with an energy filter and a special aperture to obtain almost noise‐ and background‐free diffraction patterns.^[^ ^23^ ^]^ By this innovative approach we enable nanoscale strain mapping for an ensemble of individual crystal defects contained within a 200 nm thick, single‐edge notched cantilever beam of chromium during in situ deformation in bending. It is imperative to note that while this might appear a rather limited volume, it relates favorably to micro‐/nanoelectromechanical systems structures, functional thin films, as well as representative volumes of nanostructured materials or their related fracture process zone, respectively. Importantly the same time, the sample is thick enough to mitigates surface effects common in high‐resolution TEM foils.

## 2 Results

**Figure 1:**
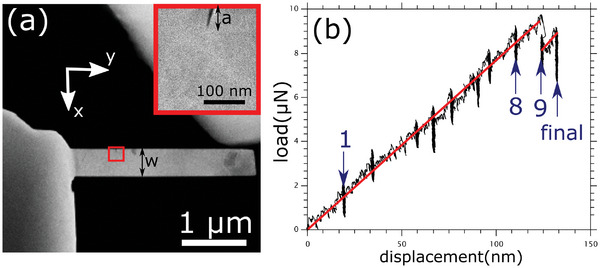
a) Annular dark‐field STEM image showing the setup used for in situ fracture testing of a Cr single crystal. A sharp notch was introduced by the electron beam to act as a stress concentrator (see insert). The width of the bending beam (W) and notch length (a) are indicated. b) The load–displacement data recorded during deformation depicts a linear elastic regime followed by a sudden load drop. Deformation was paused in between to record strain maps. The vertical streaks in the data are resultant of the load fluctuations at those points. The arrows indicate the maps presented in Figure 2, while the red straight lines serve as a guide to the eye.

For the in situ TEM deformation, well‐defined single‐leg bending beams were fabricated using focused ion beam (FIB) machining. The samples were cut from a Cr single crystal oriented along the (00$\bar{1}$)[100] (see Figures S1 and S3, Supporting Information). After preparation of the bending beam, the sample was annealed in the TEM to remove FIB induced surface‐near defects and reduce the dislocation density to bulk levels. The annealing treatment causes the implanted Ga^+^ ions to diffuse to the surface of the bending beam, allowing to measure unmodified materials parameters.^[^ ^24^, ^25^, ^26^ ^]^ **Figure** **1**a shows an annular dark‐field STEM image of the experimental setup used for the in situ deformation. A Hysitron PI‐95 picoindenter running feedback loop enabled displacement‐controlled experiments was used; this system permits the recording of load‐displacement data during deformation with high resolution. The nanoindenter tip can be seen at the top right of the image. A sharp notch was introduced along the (00$\bar{1}$)[100] direction (see insert in Figure 1a) using the condensed electron beam of the TEM to achieve an exceptionally sharp notch tip radius (<3 nm). Figure 1b shows the load–displacement curve obtained from the in situ experiment. Deformation was carried out at a fixed displacement rate of 1 nm s^−1^. In between, deformation was paused to record nano‐diffraction maps while keeping the sample under load. It should be pointed out that the load fluctuations observed during the pauses in Figure 1a do not stem from sample deformation, but the fact that the indenter drifts backward during this pausing period, leading to a slight unloading.

**Figure 2:**
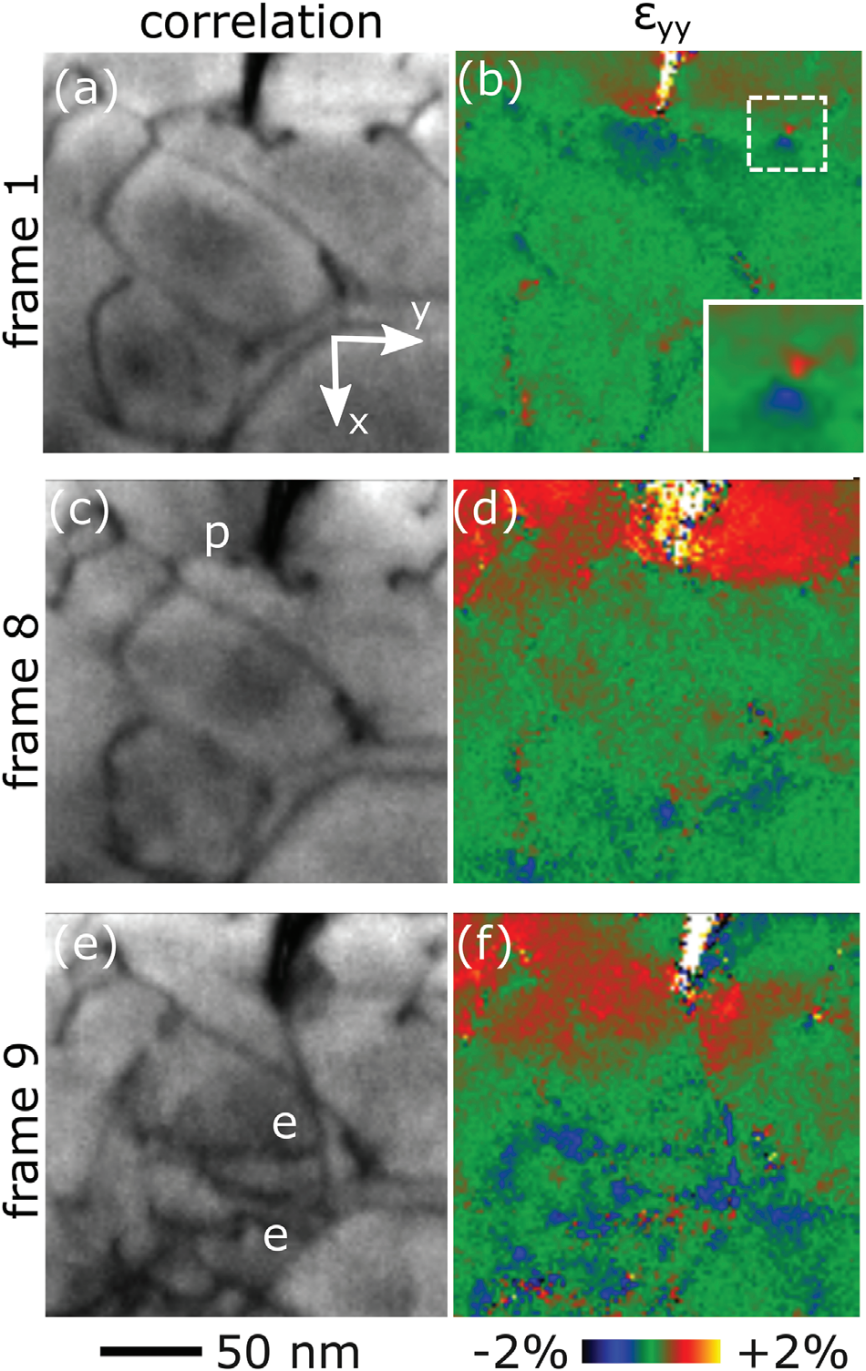
Result from in situ nano‐diffraction mapping. The mean correlation values provide a detailed visualization of the dislocation configuration on the left, while the correlated color‐coded strain ɛ_yy_ is detailed on the right. a,b) Initially, local strain is mostly visible around the dislocations. The detail shows the detailed strain field of an almost end on edge dislocation. c,d) During elastic loading the dislocation configuration remains unchanged, but there is a strong increase in tensile strain at/behind the notch tip. e,f) After the first load drop, the dislocations pinned at the notch tip (indicated p) are emitted (indicated e) and the strain field changes to a butterfly shape ahead of the notch tip.

Due to the extremely fast acquisition speed of the direct electron detector, each 4D STEM map required less than 1 min. **Figure** **2**a,b shows the results from the map acquired just after loading (denoted as 1 in Figure 1b), while the entire video is provided in Supporting Information. The local elastic strain was calculated using cross‐correlation with a template.^[^ ^27^ ^]^ To obtain robust values even during deformation, all peaks in the NBD pattern were taken into account and weighed according to the quality of the correlation. Figure 2a shows a map of the resulting mean correlation value, which is an indication for the local deviation from a perfect single crystal. In this novel visualization, dislocation lines within the whole tested volume are astonishingly well visible, in particular considering the fact that they are rather poorly imaged with standard TEM methods, which is mostly related to the large sample thickness required to ensure a bulk‐like response without unwanted near surface effects (see Figure S2, Supporting Information). The corresponding strain field ɛ_yy_, with y representing the crack‐opening direction, reveals the elastic strain field associated with the defects (see Figure 2b). Interestingly, a significant strain field is visible around the crack tip, showing a compressive strain ɛ_yy_ in front of the crack tip, indicative of crack‐tip shielding or crack bridging. The capability to map the strain around single dislocations is most evident for the edge dislocation closely aligned with the electron‐beam direction shown in the magnified insert, where the respective compressive/tensile regimes are clearly distinguished. It is important to point out that all strain maps represent raw unfiltered data. From the outstanding quality of the results in Figure 2a,b it can be concluded that the experimental setup involving an energy‐filtered direct electron detector for the first time enables the visualization of individual dislocations and their associated strain field in a large field of view of 200 × 200 nm at 2 nm resolution. More importantly, this was even accomplished during in situ deformation and using relatively thick specimens to ensure bulk behavior.

The load–displacement data recorded during the in situ experiment consists of a linear elastic regime, which is, upon further loading, eventually followed by a sudden load drop (Figure 1b). To unravel the associated nanostructural changes, the diffraction maps recorded just before and after the load drop are correlated (denoted as 8 and 9 in Figure 1b, respectively). Figure 2c,d shows the situation just before the load drop. While no significant changes in the dislocation configuration are visible, there is a strong increase in tensile strain around the crack tip, as compared to the initial unloaded state. This could be caused by a partially closed sharp crack, requiring additional load for crack opening. In any case, the strain field is influenced by the existing dislocations and remains restricted behind the crack tip. After the load drop, corresponding to the certain opening of the crack, the situation significantly changes (see Figure 2e,f). The dislocations pinned at the crack‐tip break free and two additional dislocations are emitted. Their full character is analyzed in Figure S4 (Supporting Information). To further evaluate the shielding associated with this dislocation activity in front of the crack, the stress‐intensity factor at emission, *K_e_*, is calculated in comparison to that reached after the stress drop associated with the emission process. This drop is compared to the local shielding intensity *K*_*D*, *cal*_, calculated by applying the simplified 2D back‐stress model suggested by Higashida et al.^[^ ^28^ ^]^ from the two nucleated dislocations observed in Figure 2c just after nucleation and prior to propagation, as detailed in Supporting Information. We evaluate the reduction in stress intensity as ≈0.3 MPa.m^1/2^, while the respective shielding contribution of the emitted dislocations amounts to ≈0.375 MPa.m^1/2^. Importantly, this dislocation nucleation and movement is associated with a significant change in the shape of the local strain field. Most interestingly, the strain field is no longer restricted behind the crack tip, but develops a butterfly shape around the crack tip. This particular configuration actually corresponds to the expected idealized strain field in a notched bending beam without the presence of defects.^[^ ^29^ ^]^ Therefore, it can be undoubtedly stated that the crack‐tip shielding evident in Figure 2b is diminished after crack‐tip opening and the associated emission of dislocations (Figure 2f).

**Figure 3:**
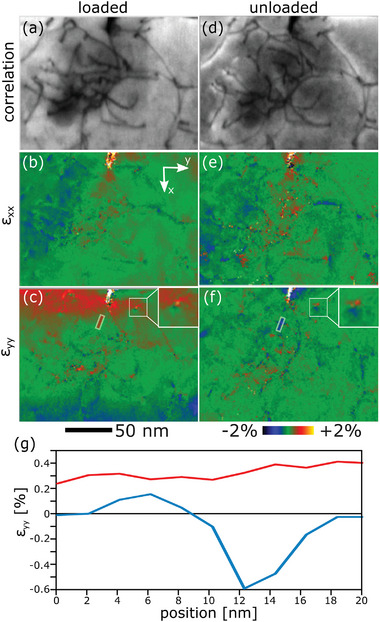
Comparison of a nano‐diffraction map acquired under load a–c) and after unloading d–f), respectively. (a,d) Only few dislocations move upon unloading, but changes are distinct in the strain fields ɛ_xx_ (b,e) and ɛ_yy_ (c,f). In unloaded condition the overall strains are reduced, in particular at the crack tip, but dislocations have a more pronounced associated local strain field. g) The strain profile (ɛ_yy_) across a dislocation is during loading (red) and after unloading (blue). The location of the profiles is indicated in b and e.

To analyze the effect of the external load on the local transient strain field around the crack‐tip and related dislocations, a large strain map was recorded under load and after unloading, respectively. The resolution of the map was kept at 2 nm to allow observation of individual dislocations, but the field of view was increased to 300 × 400 nm. **Figure** **3**a–c shows the results recorded under load (indicated in the load–displacement curve in Figure 1b with an arrow). Afterward, the sample was fully unloaded and a second map was recorded (see Figure 3d–f). Direct comparison between the respective correlation‐maps reveals only minor changes in the dislocation configuration, specifically only few short segments have moved. Contrarily, the strain state changed significantly upon unloading. This is most visible in the strain map with ɛ_yy_ showing compressive strain at the left hand side of the bending beam and tensile strain around the crack, as expected for a loaded bending beam; both features disappear upon unloading, in agreement with expectations (see Figure 3c,f). However, closer inspection shows inhomogeneous strains associated at the dislocation level. In fact, these local strain variations around individual defects are not released upon unloading, but rather tend to increase in magnitude, with most segments showing compressive strain, as can be seen by the blue contrast around the locations of dislocations in Figure 3f. To quantify this behavior, a dislocation segment in front of the crack‐tip was chosen and a line profile drawn across it (Figure 3g). The quantitative strain profile shows rather uniform tensile stresses in the loaded state (red curve in Figure 3g). After unloading, the mean strain decreases to zero, but a strong local variation emerges, indicative of a dislocation strain field, with the compressive part being dominant and pointing toward the crack‐tip (blue curve in Figure 3g). It should be mentioned that the rather low magnitude in strain results from the fact that the strain field measured here is only a 2D projection of a 3D strain field in the electron beam direction. Nonetheless, this exciting result for the first time actually details the process of dislocation crack‐tip shielding in a ductile metal.

**Figure 4:**
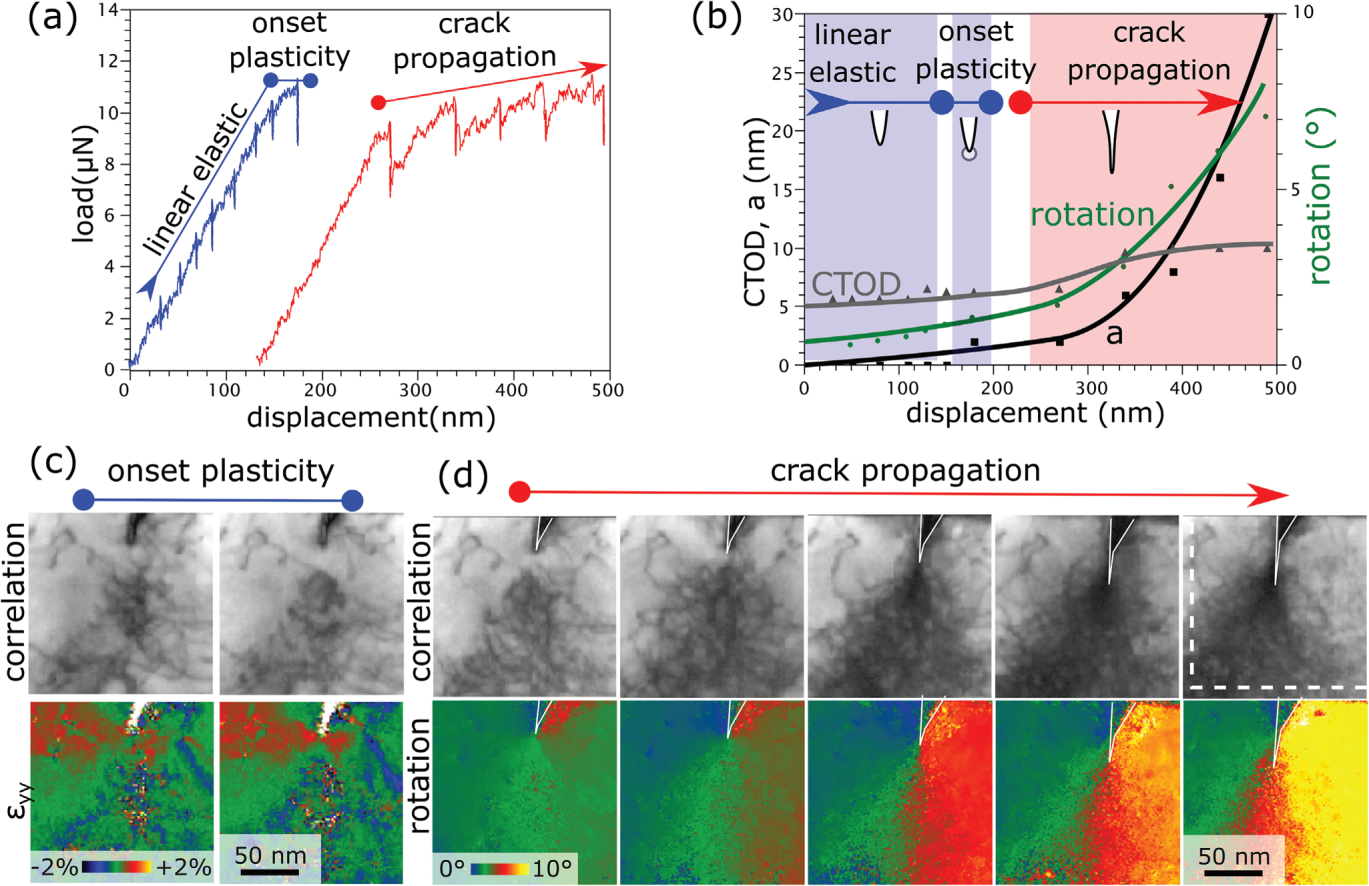
a) Load–displacement data showing elastic deformation followed by local plasticity (blue). Subsequently, the bending beam was significantly deformed to induce crack growth (red). The strain maps obtained before and after local plastic deformation are given in c). Dislocations move away from the crack tip and form a dislocation free zone ahead of the crack (indicated with a black arrow), along with an increase in magnitude of the strain field (ɛ_yy_) at the crack tip. d) Upon further plastic deformation distinctive crack tip opening is observed, accompanied by the emission of a significant amount of dislocations and connected slow crack propagation. The local lattice rotation map demonstrates the evolution of the plastic‐zone size along with the increase in rotation gradient. The crack tip is outlined in the figures to enhance correlation. Furthermore, quantitative values for the crack length, *a*, and crack‐tip opening displacement, CTOD, along with the rotation angle extracted from these in situ 4D STEM maps are given in b).

In a consecutive step, increasing deformation was applied to the same specimen to study the crack‐tip evolution all the way from elastic loading via first dislocation nucleation to fully developed crack propagation. **Figure** **4**a shows the corresponding load–displacement data. The second (re‐)loading exhibits again a linear regime, followed by the onset of plasticity (blue data in Figure 4a). As before, the deformation was carried out under displacement control and paused in between to record diffraction maps at an applied stress state before the sample was unloaded. In a final third loading step, the specimen was deformed to a displacement of around 400 nm, causing significant plastic deformation at the crack‐tip and subsequent crack growth (red curve in Figure 4a).

Figure S7 (Supporting Information) shows the maps obtained during linear elastic loading, where no significant changes in the dislocation configuration are visible; only a few segments move due to local stresses. In analogy to the results from Figure 2, high tensile strains can be evidenced around the crack tip, increasing with increasing deformation. Just at the end of the linear elastic regime, the movement of individual dislocations away from the crack‐tip is observed, in concert with an increasing extent of the crack‐tip strain field. This again underlines the intricate interplay between individual crystal defects and local strain field around the crack.

In contrast, significant structural changes can be observed when comparing the strain map obtained before and after the deviation from linear elasticity, denoted as onset of local plasticity in Figure 4a. Figure 4c shows the corresponding correlation and strain maps. The most evident feature is the evolution of a dislocation‐free zone ahead of the crack tip, as first reported for shear cracks by Ohr and coworkers,^[^ ^19^, ^30^, ^31^ ^]^ as well as the localization of the majority of dislocations into a V‐shaped zone ahead of the crack tip.

The results shown in Figure 4 also agree with previous observations for mode I cracks that have been explained by the fact that dislocations repelled from the crack‐tip act as barriers to successive dislocation emission.^[^ ^32^ ^]^ Affirmative, the strain map shows slight compressive strain exerted by the dislocations, while a high tensile strain prevails around the crack tip.

Upon further plastic deformation (indicated as crack propagation in Figure 4a), a distinct crack‐tip opening is observed, accompanied by the emission of dislocations and eventually some crack extension (Figure 4d). Apart from few dislocations that reside far from the crack‐tip and thus remain unaffected from the strain field, all dislocations concentrate in a V‐shaped zone that is always arranged in front of the crack tip. Due to the high density of defects and the large deviation from elasticity, the deformation is best described using the local lattice rotation obtained from the strain mapping. The resulting color‐coded maps demonstrate the evolution of the plastic‐zone size along with the increase in rotation gradient. Upon further deformation the apex of the plastic zone moves along with the crack tip. The crack (outlined in Figure 4d) manifests itself in the plot as the line displaying a jump in local rotation. To link the local deformation with common elastic–plastic fracture descriptors, crack growth (Δ*a*), crack length (*a*) and crack‐tip opening displacement (CTOD) are correlated to the rotation for each strain map in Figure 4b. The onset of crack propagation, manifested by an increase in crack length, can be linked to a strong increase in local rotation that is associated with the emission of dislocations. Concomitant with this strong rotation increase, the CTOD increases. With increasing crack propagation, the rotation and local dislocation density further increase, but the CTOD remains constant. These results provide a clear indication that the local plasticity, reflected by a very high density of dislocations, acts to prevent catastrophic fracture during crack propagation.

## 3 Discussion

Our results highlight the importance of local defects in controlling ductile fracture, which makes it a challenge to predict failure.^[^ ^9^ ^]^ Therefore, in order to reduce the dependence on empirical material testing, improved physical descriptions are required. Here we present a real time demonstration that the toughening of metals and alloys can result from the interplay between stimulated dislocation emission and high stresses hindering their easy propagation. To precisely balance plastic energy dissipation and crack‐tip shielding, transient local stresses around individual dislocations have to be measured. For example, twin boundaries or dislocation networks have been shown to enhance toughness.^[^ ^33^ ^]^ Local strain data in this study have permitted the quantification of the stresses around selected microstructural features and thus can enable the prediction of the strengthening and toughening associated with a given microstructure.

A comparison of the respective values of the stress intensities for atomic bond breaking at a crack tip, *K*_c_, with that for dislocation emission, *K*_e_, the latter which we have been able to directly characterize in real time in the present study, provide the fundamental basis for ideally brittle versus ductile fracture. However, this concept can also be used to predict what materials are likely to display some degree of ductility and hence damage‐tolerance. This was originally modeled in terms of the empirical Pugh ratio, defined as the ratio of the shear to bulk modulus—a low shear modulus was reasoned to promote dislocation motion whereas a high bulk modulus was considered to inhibit the opening of cracks.^[^ ^34^ ^]^ High Pugh ratios, typically exceeding 0.4 – 0.6, were therefore reasoned to signify brittle behavior. More recently, direct calculations of the values of *K*_c_ and *K*_e_, following Rice and Thomson,^[^ ^6^ ^]^ have been used for the same purpose, where the ratio *K*_e_/*K*_c_ exceeding 1 would signify a brittle material.^[^ ^35^ ^]^ Both techniques have been recently used to seek out optimal compositions in BCC refractory high‐entropy alloys.^[^ ^35^, ^36^ ^]^ As there are an almost unbounded number of such multiple principal element alloys for potential high‐temperature use, these techniques can provide an estimate of which compositions may display ductility as well as high strength, which is what is required for damage‐tolerant materials.

For Cr studied herein, using *γ* = 2.3 J m^−2^ and *E* = 294 GPa, we can estimate *K*_griffith_ =$\sqrt{2\gamma E}$−1.2 MPa m^1/2^.^[^ ^37^ ^]^ This value is a factor of 1.33 lower than the value for *K_e_* =  1.59 MPa m^1/2^ measured in the present experiments. To rationalize this, it is important to consider that the notch tip radius influences the fracture toughness, as for example addressed in detail in the work of Fischer & Beltz^[^ ^41^ ^]^ using an analytical dislocation mechanics framework. In the current work, an extremely sharp notch was introduced using e‐beam notching, resulting in a radius of around 10 times the Burgers vector (*b* = 0.204 nm). The notch length (*a* = 55 nm) corresponds to 270 times the Burgers vector. Fischer & Beltz^[^ ^41^ ^]^ have compared the critical energy release rates of dislocation nucleation for various crack lengths and crack tip radii. For a = 270*b*, increasing the notch tip radius from an ideally sharp (radius = 0.0001*b*) to the present case (radius = 10*b*) increases the critical energy release rate by a factor of around 1.69. As the critical energy release rate scales with *K* ^2^, it can be concluded that in this frame the fracture toughness *K* would increase by a factor of ≈1.30 as a result of the notch tip radius. This is in good accordance with the factor of 1.33 deduced above from relating the fracture toughness expected from a minimum surface energy Griffith‐like picture to the experimentally determined fracture toughness value. Thus, this detailed analysis additionally highlights the importance of using very sharp notches in small scale materials testing. It should be pointed out that the notch tip radius is much more critical in the failure of rather brittle materials, while materials exhibiting certain crack tip plasticity will anyways blunt the crack tip progressively with ongoing crack tip dislocation nucleation events.

To put these small scale experiments in context with macroscopic fracture experiments, it is important to note that sample size has a significant effect on material strength in micron and sub‐micron dimensions. This can potentially also affect resultant fracture toughness, most likely for cases where movement of existing truncated dislocations contribute to crack tip shielding. Furthermore, plastic deformation behavior can be significantly altered due to dislocation starvation promoted by nearby surfaces, as typically observed in small scale FCC metals with a low lattice friction,^[^ ^38^ ^]^ where one would expect the dislocation shielding contribution to be reduced by near surface annihilation of dislocations. Interestingly, during the present fracture experiment of a 200 nm thick Cr bending beam, we do not observe dislocation starvation, but dislocations are generated near the crack tip and remain within the rather thin specimen, which makes the fundamental processes more bulk‐like. This indicates that relatively thick bending beams with an extremely sharp notch radius might offer a better geometry for small scale materials testing in the TEM. Nonetheless, care should be taken when extrapolating to bulk behavior.

## 4 Conclusion

In conclusion, we have demonstrated the importance of both, pre‐existing and crack‐tip generated dislocations for the fracture behavior of a rather ductile Cr bending beam. Nanoscale strain mapping under load reveals that the dislocations provide a local strain accommodating the strain field at the crack tip. Therefore, we can conclude that toughening does not only originate from their ability to blunt the crack tip, but also to accommodate the local strain field at the crack tip. In essence, it is the complex interplay between dislocations and the local stress field that governs ductile fracture behavior. The present work shows that advancements in experimental techniques enable the direct quantification of this interplay on the vital level of individual dislocations during in situ deformation, even in relatively thick TEM specimens that approach realistic bulk dislocation configurations and minimize surface effects. The potential to quantify deformation on the most fundamental level will not only yield viable input for simulations but can also aid the design and search for novel damage‐tolerant materials.

## 5 Experimental Section

Single edge‐notched cantilever bending beams were prepared using FIB machining operating at 30 kV from single crystalline chromium. The investigated beam (see Figure 1) had a thickness of *B* = 200 nm and a width of *W* = 380 nm. The distance between notch and loading point was *L* = 1450 nm. The length of the very sharp (<3 nm) notch was *a* = 55 nm. TEM investigations were carried out on a Thermo Fischer TITAN TEM operated at 300 kV. To induce the sharp notch into the beams, the electron beam was condensed to a small spot mode and moved using beam shift.^[^ ^39^ ^]^ The bending beams were subsequently annealed in the TEM using a Gatan Model 652 heating holder for 90 min at ≈900 °C to significantly reduce the dislocation density. In situ TEM experiments were conducted with a Hysitron Picoindenter PI‐95. Feedback loop‐enabled displacement‐controlled experiments were performed at a loading rate of 1 nm s^‐1^. The experiments were paused without releasing the load to record the strain maps. More details are given in Supporting Information.

Diffraction mapping was performed with a direct electron detector (Gatan GIF Continuum K3 System) operating in continuous electron counting mode and recording 200 frames s^−1^. Zero loss energy filtering was carried out using a 20 eV slit. For the 4D‐STEM NBD mapping, STEM mode with a convergence angle of 1.5 mrad was used, resulting in a probe size less than 1 nm, compared to a strain map resolution of 2 nm. At each probe position of the STEM image a full diffraction pattern (1024 × 1024 pixels, after binning) was recorded. Custom code written as plugin for DigitalMicrograph was used for calculating strain maps from the diffraction patterns.^[^ ^27^ ^]^ Cross‐correlation with a template was used taking into account all diffracted peaks. For calculating the strain tensor, the diffraction peaks were weighted according to the correlation value obtained from the cross‐correlation. A custom bullseye condenser aperture was used to improve accuracy of the peak‐registration through cross‐correlation peaks,^[^ ^40^ ^]^ see Figure S6 (Supporting Information).

## Conflict of Interest

The authors declare no conflict of interest.

## Supporting information

Supporting Information

Supplemental Video 1

## Data Availability

The data that support the findings of this study are available from the corresponding author upon reasonable request.
