# Supplementary material for: Strain Field Around Individual Dislocations Controls Failure
Source: Small Methods. 2024 Sep 6;8(12):2400654. doi: 10.1002/smtd.202400654 (PMC11671849; doi:10.1002/smtd.202400654)
Supplement: Supplementary file 1 — Supporting Information [file SMTD-8-2400654-s001.docx]

**Supporting Information**

Strain field around individual dislocations controls failure

Christoph Gammer^1,*^, Inas Issa^2^, Andrew M. Minor^3,4^, Robert O. Ritchie^4,5^, Daniel Kiener^2,*^

^1^ Erich Schmid Institute of Materials Science, Austrian Academy of Sciences, Jahnstrasse 12, A-8700 Leoben, Austria.
^2^ Department Materials Science, Chair of Materials Physics, Montanuniversität Leoben, Jahnstrasse 12, A-8700, Austria.
^3^ National Center for Electron Microscopy, Molecular Foundry, Lawrence Berkeley National Laboratory, Berkeley, CA 94720, USA
^4^ Department of Materials Science & Engineering, University of California, Berkeley, CA 94720
^5^ Materials Sciences Division, Lawrence Berkeley National Laboratory, Berkeley, CA 94720

* Corresponding authors: christoph.gammer@oeaw.ac.at, daniel.kiener@unileoben.ac.at

**1. Methods**

**Preparation of the bending beams**

The starting material was a high purity Cr single crystal. Wedges were prepared by wire cutting followed by grinding and finally electrochemical etching. Miniaturized nanobending beams were machined by a focused ion beam (FIB) using a FIB LEO 1540XB (Zeiss, Oberkochen, Germany) operating at 30 kV. A single edge notched cantilever bending test geometry was chosen. The bending beams were clamped from one side, as shown in **Fig. S1**, reflecting the most common fracture experiment geometry used at small scales^[1–3]^*.* This setup is tolerant against misalignment between nanoindenter tip and sample surface. Also, the free end avoids residual stresses that could reimpose in double clamped bending beams^[4]^.


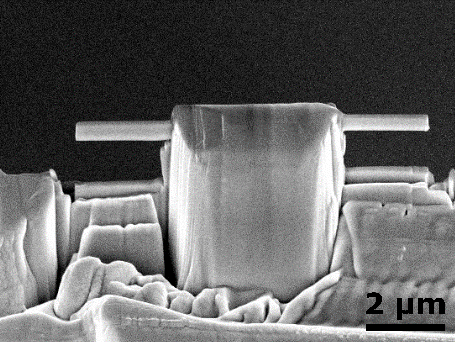


**Figure S1:** Inclined SEM image of the nanoscale bending beams.

It is well known that FIB machining creates near surface knock on damage in the sense of crystal defects, amorphization or Ga implantation. Using the SRIM software^[5]^, we assess the Ga implantation depth for the present conditions as 4.3 nm. To reduce the effects of this FIB damage, an additional annealing process was conducted. The annealing was performed for 90 minutes at ~900°C in the TEM, *i.e*., under high vacuum conditions using a double tilt heating sample holder, model 652, tantalum® from GATAN. Due to a lack in diffusion data for the Cr-Ga system, it remains challenging to estimate exact diffusion lengths, but this temperature of 0.54 *T*_m_ (melting temperature) and annealing durations in the range of an hour have been shown to be suitable to reduce crystal defects such as dislocations and FIB induced amorphization layers in nanoscale samples for various metals of differing crystal structure ^[25,6]^ or semiconductors^[39]^. Bright field TEM images of the bending beam after annealing confirm that that the sample is clean and single crystalline, but a few dislocation segments remained in the annealed beam (**Fig. S2**), in agreement with expectations for the dislocation density of a well annealed single crystal^[6]^.

Notching of the bending beams was carried out in a Thermo Fischer Titan TEM operating at 300 kV. At high magnification the electron beam was focused to a small spot (few atomic columns in width) and moved into the specimen. Notches made this way are very sharp with a radius that varies from few atomic columns to ≤3 nm^[39]^, making them comparable to nominally atomically sharp pre-cracks obtained by fatigue-crack growth for bulk fracture toughness testing. Also note that this notching was done after annealing, inside the TEM right before mechanical testing using the focused electron beam, thus no FIB was used to introduce the crack to ensure the absence of Ga at the crack tip.


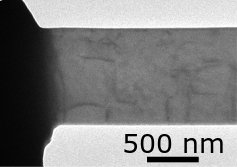


**Figure S2:** Prior to the deformation the bending beam was imaged using conventional bright-field TEM. The dislocations are hard to discern due to the high sample thickness of ~200 nm.

**Sample characterization**

The specimen thickness *B* is measured in the SEM as *B* = 200 nm. The width is determined in the TEM and corresponds to *W*= 380 nm. The notch length was measured to be *a*= 55 nm. The length *L* corresponds to the distance from the notch to the loading point and was measured to be *L*= 1950 nm, also determined in the TEM. As seen from the diffraction information, the sample was a single crystal oriented along [010], with notches along (100)<100>, as shown in **Fig. S3**.


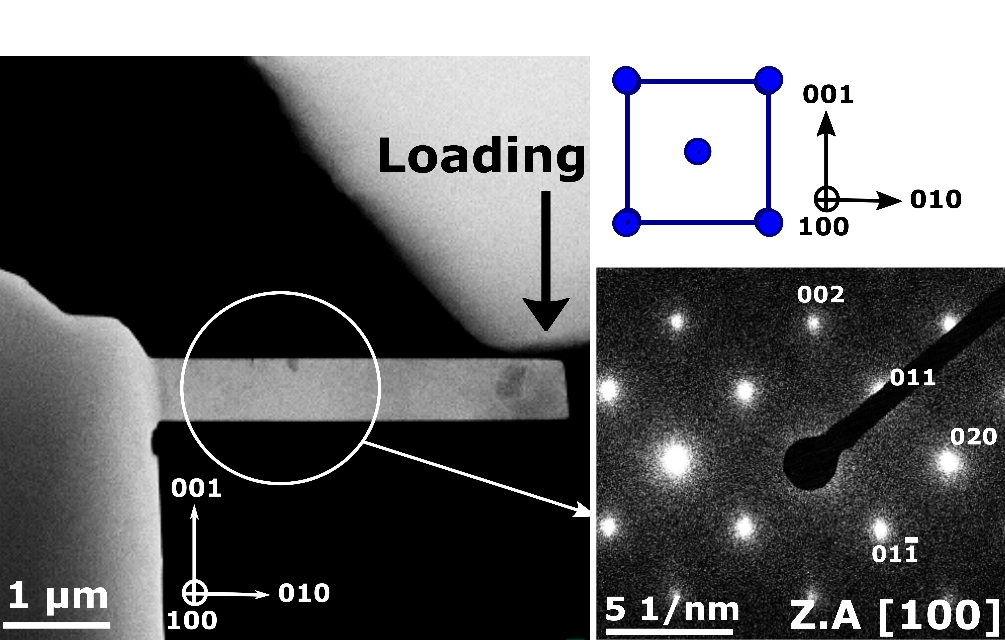


**Figure S3:** STEM image of the experimental setup showing the specimen and the respective loading direction of the diamond tip. The corresponding diffraction pattern of the specimen shows that it is a single crystal Cr with the long axis being oriented along [010].

**Dislocation analysis**

Deformation in body-centered cubic (BCC) metals, such as chromium, is well documented to occur commonly along slip planes {110} and slip directions <111>; hence, 12 ½<111>{110} slip systems exist. Note that there are other slip systems possible^[7]^, but since they do not align with our observations, we refrain from detailing them here.

In order to proceed and characterize emitted dislocations in the notch zone, we first estimated the most possible active slip systems in such a crystal orientation with respect to the tensile stress direction on the notch tip during the test, as presented in **Fig. S4a**.


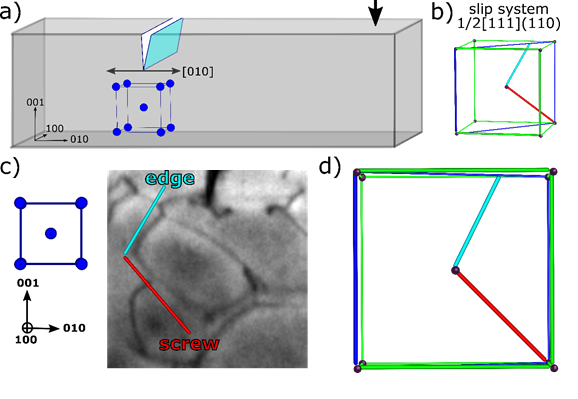


**Figure S4:** a) Schematic of the experimental setup showing the unit cell orientation in the specimen and the tensile crystallographic direction. b) Model depicting screw and edge dislocations vector lines of the ½[111](110) slip systems. c) STEM image of the notch area after few dislocations’ emission. d) Projection of the slip system model to equivalent experimental orientation.

Accordingly, we calculated the Schmid factors for each slip system, where the Schmid factor *S* is given by *S* = cos (φ) cos(λ). Here φ is the angle between each specific slip plane from the family {110} and the tensile direction [010], and λ is the angle between each specific slip direction from the family <111> and the tensile direction [010]. From the Schmid factors calculations in **Table S1**, we find that that 8/12 slip systems (highlighted) are equally possible to be activated in such an experimental configuration setup and crystal orientation with *S*= 0.4. It should be noted that this is only a simplified approach assuming uniaxial loading, and more advanced correlative experimental-computational approaches would enable an in-depth assessment if required^[8]^.

**Table S1: *Schmid factors calculations***

| **Slip system** | **φ** | **λ** | ***S* = cos (φ). cos(λ).** |
| --- | --- | --- | --- |
| ½[$\bar{1}$11](101) | 90° | 54.74° | 0 |
| ½[$\bar{1}\bar{1}1]$(101) | 90° | 54.74° | 0 |
| ½[111](10$\bar{1}$) | 90° | 54.74° | 0 |
| ½[1$\bar{1}$1](10$\bar{1})$ | 90° | 54.74° | 0 |
| ½[$\bar{1}\bar{1}$1](011) | 45° | 54.74° | 0.4 |
| ½[1$\bar{1}$1](011) | 45° | 54.74° | 0.4 |
| ½[$\bar{1}$11](01$\bar{1}$) | 45° | 54.74° | 0.4 |
| ½[111](01$\bar{1})$ | 45° | 54.74° | 0.4 |
| ½[$1$11](110) | 45° | 54.74° | 0.4 |
| ½[1$\bar{1}$1](110) | 45° | 54.74° | 0.4 |
| ½[111](1$\bar{1}$0) | 45° | 54.74° | 0.4 |
| ½[$\bar{1}\bar{1}$1](1$\bar{1}$0) | 45° | 54.74° | 0.4 |

For the present work, observed dislocations around the notch in the STEM image in Fig. S4c are characterized by comparison with crystallographic models of dislocations projected along this crystal orientation. These dislocation lines favorably fit to the screw and edge dislocations of the slip system ½[111](110) as shown schematically in Fig. S4b, while the projected view corresponding to the experiment is shown in Fig. S4d.

**Load-drop evaluation and related analysis**


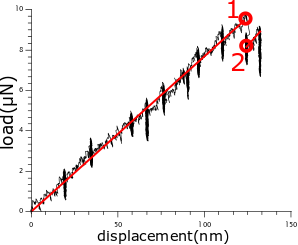


**Figure S5:** Load-displacement curve highlighting load drop between points 1 and 2.

To calculate the instantaneous stress-intensity factors at the observed dislocation emission event (*K*_e_) (see Fig. 2e) and after the respective load drop (*K*_2_), we used the following *K*-solution for the single-edged notched cantilever beam^[2]^:

$$K_{e,2}=\frac{F_{e,2}*L}{B*W^{3/2}}*f(\frac{a}{W})$$

where, *F*_e_ = 9.8752 µN is the load associated to dislocation emission (point 1 in **Figure S5**) and *F*_2_ = 8.33 µN the load after the load drop (point 2 in Fig. S5). The values for width (*W*), notch length (*a*), thickness (*B*) and length (*L*) are provided in chapter Sample characterization, while $f(\frac{a}{W})$is the geometry factor depending on the ratio *(a/W)* calculated for a single edge notched cantilever bending test, as given by the following relationship:^[3]^

| $f\left( \frac{a}{W} \right) =4\frac{\left\{ (3\left( \frac{a}{W} \right)^{0,5}\left( 1,23-\left( \frac{a}{W} \right)\left( 1-\frac{a}{W} \right) \right))(-6.09+13.96\left( \frac{a}{W} \right)-14,05\left( \frac{a}{W} \right)^{2}) \right\}}{2{(1+2\left( \frac{a}{W} \right))(1-\frac{a}{W}))}^{1,5}}$ |
| --- |

The respective instantaneous stress-intensity factors are evaluated to amount to: $K_{e}=1.59 MPa.m^{1/2}$ and $K_{2}=1.33 MPa.m^{1/2}$, respectively. Thus, from this load-drop, we can associate a decrease in the stress-intensity factor at the crack tip of the specimen ($K_{e}- K_{2})\approx0.3 MPa.m^{1/2}$ to be connected to this observed dislocation emission event (compare Fig. 2c to Fig. 2e).

The local shielding intensity $K_{D, cal}$ caused by the presence of the two dislocations which were emitted from the notch tip (Fig. 2c) is calculated by applying the simplified 2D back-stress model developed by Higashida *et al*.^[28]^, *viz*:

$$K_{D,cal}=\sum\left\{ \frac{3\mu b_{i}}{(1-\nu){(2\pi r_{i})}^{1/2}}\cos\left( \frac{\theta}{2} \right)sin(\varphi) \right\}$$

with *μ* (120 GPa) being the shear modulus, *b* (0.204 nm) the Burgers vector, *ν* (0.22) the Poisson’s ratio of Cr, $r_{i}$ the distance of each dislocation to the first dislocation at the crack tip, and $\theta$ the angle between slip plane (110) and crack direction, taken to correspond to [001]. Upon conducting this analysis, we obtain $K_{D, cal}$~ 0.375 $MPa.m^{1/2}$ as shielding contribution of the two newly nucleated dislocations, which is convincingly close to $K_{e}- K_{2}$ assessed from the analysis of the mechanical specimen response.

**Nano-diffraction patterns recorded using special setup**


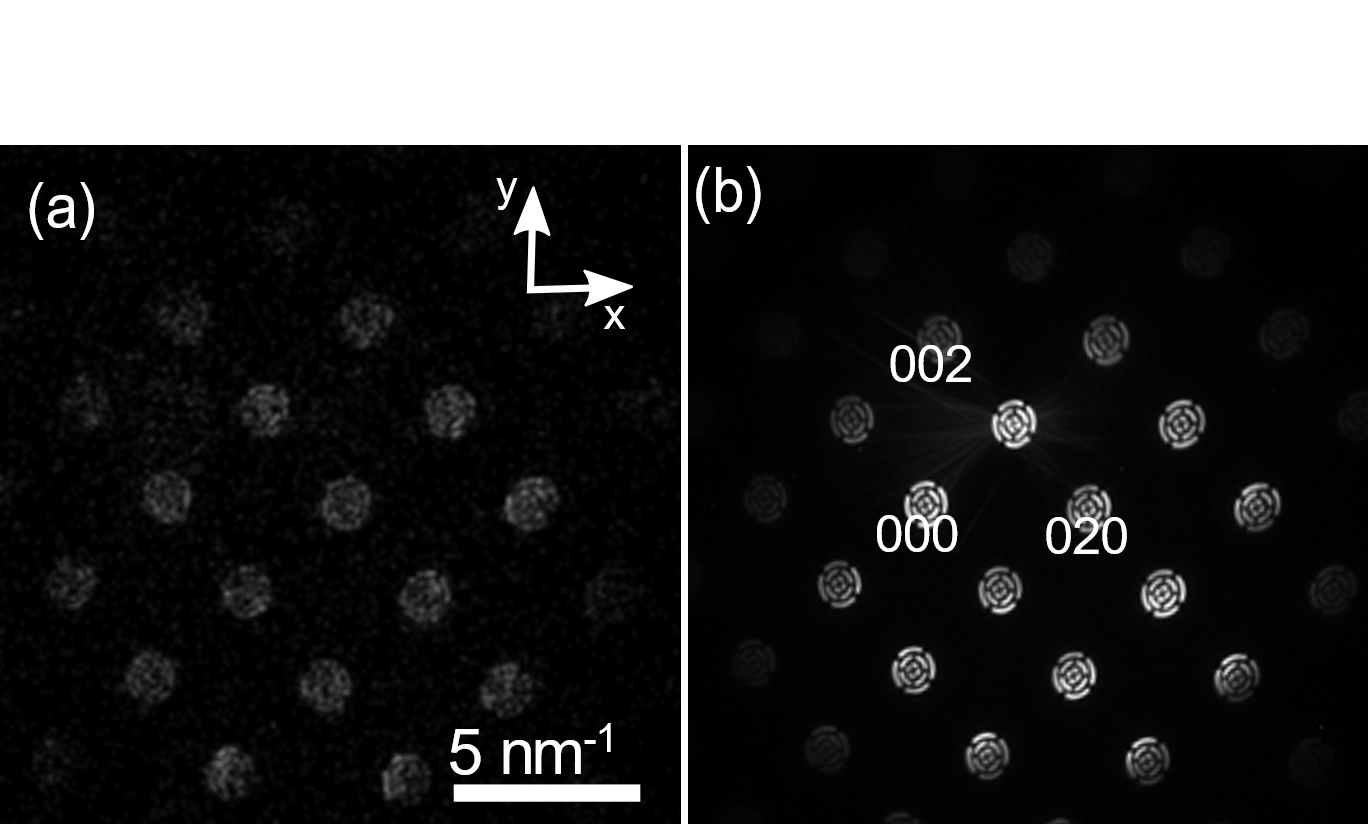


**Figure S6:** (a) Single nanobeam diffraction pattern recorded using an energy-filtered direct electron detector. All counts originate from individual electrons. No significant background is visible due to the zero-loss filtering. (b) Sum of several nanobeam diffraction patterns. A special bullseye condenser aperture used to enhance autocorrelation becomes visible^[40]^.

**2. Results and discussion**

**Nano-diffraction map acquired during second elastic loading**

*
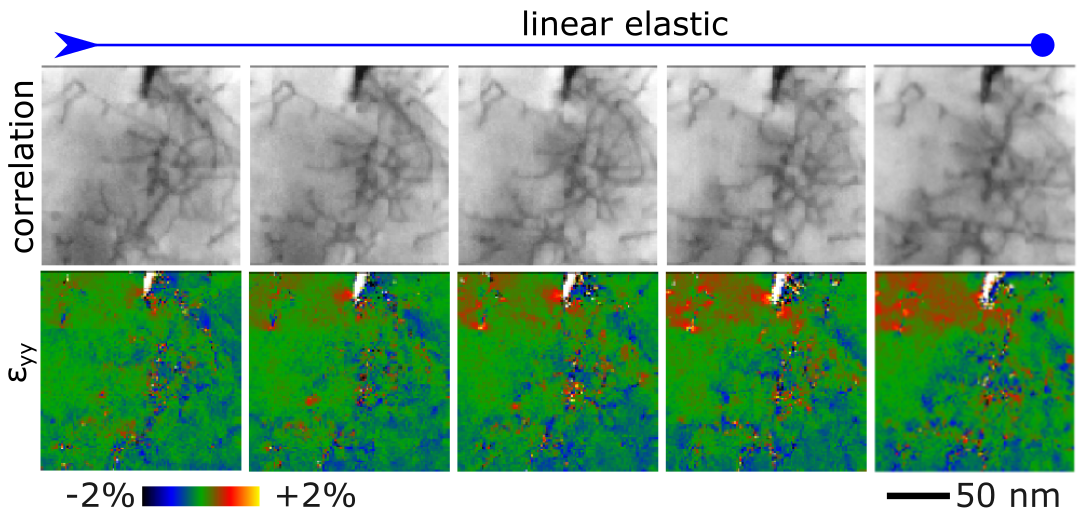
*

**Figure S7:** Results from the nano-diffraction map recorded during the second elastic loading (denoted as linear elastic in **Fig. 4a** of the main manuscript). The correlation map along with the strain field (ε_yy_) is shown.

**3. Supplementary movie**

**Video S1:** Entire video corresponding to Fig. 2a.

References

[1] B. N. Jaya, J. M. Wheeler, J. Wehrs, J. P. Best, R. Soler, J. Michler, C. Kirchlechner, G. Dehm, *Nano Lett.* **2016**, *16*, 7597.

[2] K. Matoy, H. Schönherr, T. Detzel, T. Schöberl, R. Pippan, C. Motz, G. Dehm, *Thin Solid Films* **2009**, *518*, 247.

[3] S. Wurster, C. Motz, R. Pippan, *Philos. Mag.* **2012**, *92*, 1803.

[4] B. N. Jaya, C. Kirchlechner, G. Dehm, *J. Mater. Res.* **2015**, *30*, 686.

[5] J. F. Ziegler, M. D. Ziegler, J. P. Biersack, *Nucl. Instruments Methods Phys. Res. B* **2010**, *268*, 1818.

[6] D. Kiener, Z. Zhang, S. Šturm, S. Cazottes, P. J. Imrich, C. Kirchlechner, G. Dehm, *Philos. Mag.* **2012**, *92*, 3269.

[7] C. R. Weinberger, B. L. Boyce, C. C. Battaile, *Int. Mater. Rev.* **2013**, *58*, 296.

[8] D. Steinberger, I. Issa, R. Strobl, P. J. Imrich, D. Kiener, S. Sandfeld, *Comput. Mater. Sci.* **2023**, *216*, 111830.
